# Quercetin and Its Anti-Allergic Immune Response

**DOI:** 10.3390/molecules21050623

**Published:** 2016-05-12

**Authors:** Jiri Mlcek, Tunde Jurikova, Sona Skrovankova, Jiri Sochor

**Affiliations:** 1 Department of Food Analysis and Chemistry, Faculty of Technology, Tomas Bata University in Zlín, Vavreckova 275, CZ-760 01 Zlín, Czech Republic; skrovankova@ft.utb.cz; 2 Institute for Teacher Training, Faculty of Central European Studies, Constantine the Philosopher University in Nitra, Drazovska 4, SK-949 74 Nitra, Slovakia; tjurikova@ukf.sk; 3 Department of Viticulture and Enology, Faculty of Horticulture, Mendel University in Brno, Valticka 337, CZ-691 44 Lednice, Czech Republic; sochor.jirik@seznam.cz

**Keywords:** quercetin, flavonoids, immune response, anti-allergic effect, anti-inflammatory properties

## Abstract

Quercetin is the great representative of polyphenols, flavonoids subgroup, flavonols. Its main natural sources in foods are vegetables such as onions, the most studied quercetin containing foods, and broccoli; fruits (apples, berry crops, and grapes); some herbs; tea; and wine. Quercetin is known for its antioxidant activity in radical scavenging and anti-allergic properties characterized by stimulation of immune system, antiviral activity, inhibition of histamine release, decrease in pro-inflammatory cytokines, leukotrienes creation, and suppresses interleukin IL-4 production. It can improve the Th1/Th2 balance, and restrain antigen-specific IgE antibody formation. It is also effective in the inhibition of enzymes such as lipoxygenase, eosinophil and peroxidase and the suppression of inflammatory mediators. All mentioned mechanisms of action contribute to the anti-inflammatory and immunomodulating properties of quercetin that can be effectively utilized in treatment of late-phase, and late-late-phase bronchial asthma responses, allergic rhinitis and restricted peanut-induced anaphylactic reactions. Plant extract of quercetin is the main ingredient of many potential anti-allergic drugs, supplements and enriched products, which is more competent in inhibiting of IL-8 than cromolyn (anti-allergic drug disodium cromoglycate) and suppresses IL-6 and cytosolic calcium level increase.

## 1. Introduction

The expansion of allergic diseases has been extended during last three decades all over the world. Different changes in environmental factors (sensitizers such as indoor and outdoor allergens, air pollution, and various infections) may contribute to this problem [1]. Dietary alterations relating to nutritional changes of foods and their amount could be one of the factors that cause increase and worsening of allergic symptoms too. The interaction of environmental and genetic factors with the immune system can thus lead to the development of allergic diseases [2]. Mainly respiratory, skin, and food allergies are included in the inherent allergic problems. The immune system reacts quite sensitively to familiar substances that are, after re-exposure, sensed as allergens. This leads to a massive secretion of allergy-related mediators that could generate allergic symptoms [3].

Therapy with different synthetic agents or drugs could cause certain patients various adverse side effects. Phytochemicals, such as flavonoids, polysaccharides, lactones, alkaloids, diterpenoids and glucosides, are naturally present in many plant foods. These compounds have been reported to be responsible for immunomodulating and anti-inflammatory properties [4]. Dietary polyphenols as natural therapies have been more frequently studied with regard to major allergic diseases such as atopic eczema, food allergy and asthma [5,6,7]. Therefore, the attention concerning the compounds with anti-allergic effect and anti-inflammatory properties has been presently focused on flavonoids, especially quercetin. Its anti-inflammatory and anti-allergic properties have been proven in the treatment of respiratory and food allergies [8,9,10].

Quercetin belongs to the most frequently studied flavonoids that is, together with kaempferol, the most ubiquitous in plant foods although they are generally presented at relatively low concentrations of 15–30 mg/kg FW (fresh weight) [11] except several vegetable varieties with extensive content, such as onions and shallots. Quercetin and its glycosylated forms represent 60%–75% of flavonoid intake [12]. Generally, quercetin occurs in natural plant sources such as various types of vegetable (onions, broccoli, and peppers) [13,14,15], caper fruits [16], different kinds of fruits (apples, various berries, and grapes) [17,18,19], herbs (dill) [20], and some types of tea [21] and wine [22,23].

Besides other natural forms of quercetin, its plant extract is the main ingredient of many potential anti-allergic drugs, supplements and enriched products with quercetin, such as functionally enhanced white and red wines [24]. Quercetin is also a promising component that can prevent lifestyle related diseases [25]. Quercetin extracts are now largely used as a nutritional supplements and curative ingredients for many illnesses such as diabetes related with obesity and circulatory dysfunction, including inflammation, as well as moods troubles [26]. Quercetin exhibits similar anti-allergic potential as a Chinese herbal formula (Food Allergy Herbal Formula) that has been related with blocking of anaphylaxis to peanuts in mouse models [27].

## 2. Quercetin, Its Structure and Main Sources

**Figure 1:**
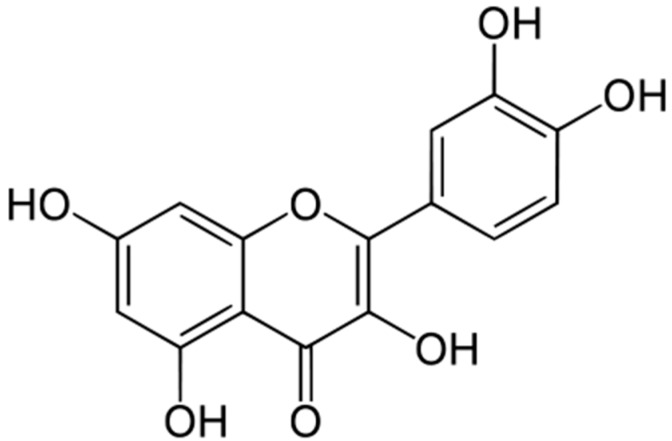
Chemical structure of quercetin.

Quercetin (3,3′,4′,5,7-pentahydroxyflavone) (Figure 1) is one of the most abundant dietary flavonoids and belongs to the flavonols subgroup. Flavonols (C6-C3-C6 polyphenols) have two hydroxylated benzene rings, A and B. Flavonols differ in the number and type of substitution in the B ring, where quercetin is dihydroxylated in positions 3′ and 4′.

Quercetin is present in plants in many different glycosidic forms. It is usually found in conjugated forms with sugars such as glucose, galactose and rhamnose [28]. The prevalent forms are quercetin conjugated with one or two glucose molecules, such as isoquercetin; and quercetin conjugated with rutinose such as quercetin rutinoside—rutin. However, the aglycone form of quercetin occurs in much lower levels in foods. Different quercetin forms represent 60%–75% of flavonoid intake [12].

The quercetin content of plant foods differs depending on the cultivars or cultivation conditions [25,29,30]. Its content has been shown to also be dependent on light exposure [31].

Quercetin is a flavonol that is found in a considerable quantity in various vegetables such as onions and shallots that are affordable throughout the year. In many countries, onions are the main sources of dietary quercetin [32,33,34]. Onions are thus qualitatively and quantitatively the most important source of quercetin. Other vegetables, including broccoli, asparagus, green peppers, tomatoes and red leaf lettuce, could be great sources of ubiquitous quercetin, especially in the summer [25,33,35]. Fruits (apples as well as berry crops, such as strawberry, red raspberry, blueberry, cranberry and black currants), green tea and wine could also be considered abundant dietary sources [22,25,33,34,35,36,37]. In Finland, onions and apples are the best sources of this dietary flavonoid, while in the Netherlands apples rank third behind tea and onions as top sources of flavonoids [33,38].

Onions (*Allium cepa*) are one of the richest sources of flavonoids in the human diet. The main flavonoids of onions are represented by quercetin and its conjugates [39,40]. The principal forms are quercetin 4′-glucoside, quercetin dimers, 3,4′-diglucoside, and quercetin 3-glucoside, with the content in the range of 284–486 mg/kg [33,41]. The amount of quercetin in onions varies with bulb color and type [42,43], being distributed mostly in the outer skins and rings [44,45], as flavonols are mainly located in the outer epidermis of the skin, because they have a UV-protecting function [46]. Thus, the highest loss of quercetin happens when onions are peeled [40,47]. According to the part of onions and shallots that is consumed, quite various amounts and forms of quercetin are ingested. The quercetin forms of onion flesh include especially glucosides, with only minimal amounts of quercetin aglycone. The skin and outermost layers of onions, similar to shallots, encompass much more quercetin aglycone [32,48,49,50]. The forms of quercetin in shallot flesh are composed of about 99.2% quercetin glucosides and 0.8% quercetin aglycone. In dry shallot skin, the form distribution is nearly reversed—83.3% quercetin aglycone and 16.7% quercetin glucosides [51].

In apples, another great quercetin source, there are well studied antioxidant compounds such as quercetin-3-galactoside, quercetin-3-glucoside, and quercetin-3-arabinoside [33,41] in the content range of 21–72 mg/kg; quercetin-3-rhamnoside [52]; and quercetin-3-rutinoside [28]. Quercetin conjugates are present entirely in the apple peels [28,53]. Due to the presence of more antioxidants such as quercetin in the apple peel than in the flesh, the apple peels may be considered to have higher antioxidant capacity and also bioactivity [54]. In apples, other dietary quercetin glycosides such as quercetin galactosides are also found [34].

Berry crops are great representatives of quercetin glycosides, especially quercetin-3-glucoside (strawberry and red raspberry), quercetin-3-glucuronide (strawberry, red raspberry and blueberry), quercetin-3-rutinoside (strawberry, red raspberry and blueberry), qurecetin-3-rhamnoside (red raspberry, black currants, blueberry, and cranberry), quercetin-3-galactoside (blueberry, cranberry, and black currants), and quercetin-3-arabinose (cranberry) [33,34,41,55].

Also lesser known fruit species could be considered as notable sources of quercetin. Saskatoon berries (*Amelanchier alnifolia*) can be characterized by exceptionally high value of quercetin, as determined by Jurikova *et al*. [56]. The quercetin content in some cultivars was quantified from 236 to 307 mg/kg FW. Saskatoon berry fruits contain quercetin-3-galactoside as the main abundant flavonol (169.5 mg/kg FW) [57]. In six cultivars of edible honeysuckle (*Lonicera* sp.), quercetin-3-rutinoside and quercetin-3-glucoside are identified as dominated flavonols. The quercetin level is high especially in *Lonicera caerulea* var. *edulis Turcz. (‘Altaj’)* (294.1 mg/kg) [58]. Quercetin was the main flavonol detected in chokeberry (*Aronia* sp.) with the content 89 mg/kg FW [36]. In black chokeberry (*Aronia* *melanocarpa*) Määttä-Riihinen *et al.* [59] detected the overall amount of 348 mg/kg, which was the fourth highest quercetin value of 18 berries they studied from six families (*Grossulariaceae*, *Ericaceae*, *Rosaceae*, *Empetraceae*, *Elaeagnaceae*, *Caprifoliaceae*), after the amounts of three bog whortleberry (*Vaccinium uliginosum*) genotypes. Quercetin is the third most representative flavonoid in cornelian cherry (*Cornus mas*) in the amount from 120 up to 360 mg/kg [60]. Quercetin rhamnosyl hexoside and dirhamnosyl hexoside, and isoquercetin are the main flavonols, which follow after kaempferol, in the fruits of Chinese hawthorn (*Crateagus pinnatifida*) [56].

Flavonol glycosides are one of the most important groups of polyphenols besides catechins in tea. The composition of tea varies with type, variety, season, age of leaves, climate, and horticultural practices [61]. Black tea and oolong tea, both fermented tea types, have the highest content of quercetin types of flavonol glycosides (50%–52% in oolong tea and 54%–71% in black tea, respectively); green tea manufactured without the process of fermentation has a higher content of kaempferol glycosides, while quercetin represents about 18%–38% of all flavonol glycosides [62]. The predominant quercetin glycosides in black tea are quercetin glucoside, rutinoside (rutin), and galactoside; minor representatives are quercetin dirhamnoglucoside and rhamnogalactoside; and quercetin rhamnodiglucoside is present in the lowest levels. Oolong tea contains mainly quercetin glucorhamnoglucoside, rutinoside, rhamnogalactoside and quercetin dirhamnoglucoside. Quercetin rhamnogalactoside, rhamnodiglucoside and quercetin glucorhamnoglucoside are principal glycosides presented in green tea [62]. The main quercetin forms of green pu-erh tea comprise two constituents, quercetin 3-rhamnosylgalactoside and 3-glucoside. White tea has as predominant flavonol quercetin 3-glucosylrutinoside [63]. There is about 15–35-times higher content of rutin than quercetin [21].

Quercetin is synthesized in white and light red grape varieties together with other mono- and di-substituted B-ring derivatives, kaempferol and isorhamnetin [64], which is the methylated form of quercetin. Quercetin is therefore the main flavonol of white wine varieties (Chardonnay, Riesling, and Sauvignon Blanc), representing over 70% of the total flavonol content [65]; and also of some light red/rosé wine varieties (Pinot Noir, Sangiovese, and Gewürztraminer). Glucose or glucuronic acid and galactose are the main sugars in conjugated forms of quercetin in wines [19].

## 3. Antioxidant Activity of Quercetin

Quercetin is a frequently studied phenolic compound due to its known great antioxidant properties. Its flavonoid structure, 2,3 double bound in conjunction with 4-oxo bond in the C ring of quercetin, allows electron delocalization from the B ring and shows extensive resonance. This results in the significant efficiency for radical scavenging [66]. Therefore, quercetin has a structure that is responsible for higher antioxidant activity effectiveness than the structure of anthocyanins.

Quercetin seems to be one of the most powerful flavonoids for protecting the body against reactive oxygen species [4,67]. Quercetin, similar to fisetin, catechin, and myricetin, are flavonoid aglycones that have 3-OH groups and are potent inhibitors of lipid oxidation [68]. Quercetin inhibits the initiation step in chain oxidation and prevents chain propagation. This may also include the termination of a chain by the reaction of two radicals [3,8].

Endogenous antioxidant ability of quercetin modifies the range of cellular injury during the allergic damage that is caused by free radicals. Enzymes, such as repair and *de novo* ones (lipases, DNA repair enzymes, proteases and transferases), act as the third line of defense by repairing damage and reconstituting membranes [4,69].

## 4. Polyphenols and Quercetin as Effective Anti-Allergic Secondary Metabolites

Polyphenols are considered effective anti-allergy agents capable of influencing multiple biological pathways and immune cell functions in the allergic immune response. Among the most investigated plant-derived polyphenolic compounds (flavonoids), quercetin, together with resveratrol, epigallocatechol-3-gallate, and genistein, have exhibited potent effects on cellular and humoral immune functions in pre-clinical investigations [70]. The interaction of polyphenols with proteins can modulate the process of allergic sensitization and their direct effect on allergic effector cells such as mast cells inhibit mediator release, resulting in the alleviation of symptoms [3]. Polyphenols inhibit histamine release from human basophils and murine mast cells [71,72].

Intake of polyphenols such as flavones, flavone-3-ols, catechins, anthocyanidins, flavanones, procyanidins, and resveratrol can improve a skewed balance of T-helper (Th) type 1 and 2 cells (Th1/Th2) and suppress antigen-specific IgE (Immunoglobulin E) antibody formation [73].

Flavonoids are known to inhibit histamine release from human basophils and murine mast cells [71,72]. Flavonoids inhibit the release of chemical mediators; further suppress interleukin (IL)-4 and IL-13 synthesis (Th2 type cytokines) by allergen- or anti-IgE antibody-stimulated receptor-expressing cells (e.g., peripheral blood basophils or mast cells). They can also affect the differentiation of naïve glycoprotein CD4 (cluster of differentiation 4) T cells (white blood cells) due to the inhibitory effect on the activation of the aryl hydrocarbon receptor [1,74]. The inhibitory activity of flavonoids on IL-4 and CD40 ligand expression is probably related through their inhibitory action on activation of nuclear factors of activated T cells and AP-1 (activator protein-1) [1].

Flavonols extracted from plants inhibit histamine and some cytokines release from rodent basophils and mast cells. Basophils are more responsible for this balance than tissue mast cells, therefore they could be considered as the potent natural substances for allergy cure [75].

Nowadays, the attention is focused on immunomodulation and anti-inflammatory properties of quercetin such as stimulation of immune system, antiviral activity (anti-herpes virus type I), inhibition of histamine release, inhibition of nuclear factor activation (NF-κB), pro-inflammatory cytokines and leukotrienes [8,76].

Quercetin induces significant gene expression and production of Th-1-derived interferon (IFN)-g, as well as downregulating Th-2-derived IL-4 production by normal peripheral blood mononuclear cells [77]. The anti-inflammatory profile of quercetin is known to impact on the recruitment of immune cells to the skin and in preventing the development of secondary infections following disruption of the skin barrier [3].

The anti-inflammatory action of quercetin is caused by the inhibition of enzymes such as lipoxygenase, and the inhibition of inflammatory mediators. Quercetin affects immunity and inflammation by acting mainly on leukocytes and targeting many intracellular signaling kinases and phosphatases, enzymes and membrane proteins often crucial for a cellular specific function [78]. Quercetin inhibits the production and release of histamine and other allergic and inflammatory substances, possibly by stabilizing cell membranes of mast cells [79,80]. In particular, quercetin is an inhibitor of allergic (IgE-mediated) mediator release from mast cells and basophils, another type of white blood cell involved in immune reactions. Quercetin is also an inhibitor of human mast cell activation through the inhibition of Ca^2+^ influx, histamine, leukotrienes and prostaglandins release and proteinkinase activation [27]. Mast cells are influential immune cells important for the pathogenesis of allergic responses and autoimmune disorders. They also affect release of many cytokines involved in the inflammatory reactions such as IL-8 and Tumor necrosis factor (TNF) [81,82]. It is a reason why quercetin is suitable for the treatment of mast cell-derived allergic inflammatory diseases such as asthma, sinusitis, and rheumatoid arthritis [8].

The anti-allergic and anti-inflammatory properties of quercetin have been proved by several studies, in animal models and *in vitro*, as presented in Table 1.

### 4.1. Quercetin and Respiratory Allergic Diseases: In Vitro and Animal Studies

Nowadays, the most studied food products in relation to their anti-allergic properties are onions and their extracts. The anti-allergic activity (type I hypersensitivity) of different onion cultivars (eight cultivars from three geographical origins) were determined by Sato *et al*. [29] using rat mast cells (RBL-2H3 cells). Extensive variation of anti-allergic effectiveness was ascertained between the cultivars. Between the studied types, Satsuki cultivar demonstrated the highest activity due to inhibitory concentration value (IC_50_ = 89.1 mg/mL). The positive correlation (r = 0.91) was exhibited between compounds of onion extracts, such as quercetin 4′-glucoside, and anti-allergic response.

Oliveira *et al.* [94] investigated the effect of onion extract and quercetin on cytokines and on smooth muscle contraction *in vitro* and its effectiveness in a murine model of asthma. After a treatment with onion extract or quercetin, they examined a decrease in inflammatory cytokines creation, a release of tracheal rings and a lowering of cells quantity in bronchoalveolar lavage and eosinophil peroxidase in lungs.

An herbal fraction from onion bulb can inhibit histamine release and reduce intracellular calcium levels in induced rat peritoneal mast cells. Eosinophil peroxidase activity and protein amount in bronchoalveolar lavage fluid of ovalbumin-challenged mice were also inhibited [89].

The effect of quercetin on pro-inflammatory mediator release and its probable principles of action in human mast cells was investigated by Kempuraj *et al*. [81]. Human umbilical cord blood-derived cultured mast cells grown in the presence of stem cell factor and interleukin (IL)-6 were incubated for 15 min with quercetin (0.01 µM), followed by activation with anti-IgE. Release of IL-6, IL-8 and TNF-α was inhibited by 82%, tryptase release by 79%–96%, and histamine release by 52%–77% at 100 µM concentration of quercetin, Min *et al.* [84] also investigated the effect of quercetin on the expression of pro-inflammatory cytokines in human mast cell line. These cells were stimulated with phorbol 12-myristate 13-acetate (PMA) and calcium ionophore. Results of the experiment showed that quercetin decreased the gene expression and production of tumor necrosis factor-α, interleukin-1β, IL-6, and IL-8 in human mast cells. Quercetin reduced phorbol calcium ionophore-induced activation of NF-κB and p38 mitogen-activated protein kinase.

Quercetin has a significant inhibitory effect on histamine release. The effect of quercetin on histamine secretion from antigen sensitized mast cells was examined by Fewtrell and Gomperts already in 1977 at µM concentrations [86]. Quercetin had an inhibitory effect on histamine secretion mediated by antigen, but it had little effect on release induced by the ionophores. Quercetin exerts its effect after the binding of the releasing ligands and the distinction between its effect on ligand induced and A23187 induced secretion. That suggests that it influences the path of Ca^2+^ entry into the cell.

Like histamine and most cyclin-dependent kinases, quercetin restrains the *in vitro* growth of some malignant cells and thus indicates considerable anti-cancer effects. Quercetin could restrain compounds responsible for allergy response due to the inhibition action of mast cell secretion, effecting regress in the release of tryptase, monocyte chemoattractant protein 1 (MCP-1) and IL-6 [85].

Haggag *et al*. [87] tested inhibitory effect of herbs infusion (chamomile, saffron, anise, fennel, caraway, licorice, cardamom and black seed) on histamine that was relinquished from stimulated rat peritoneal mast cells by immunoglobulin E and anti-immunoglobulin E. The results of the experiment showed that the herbs infusion restrained histamine released from chemically- and immunologically-induced cells by 81% and 85%, respectively. In comparison, quercetin appears to be more effective, as it affected histamine release by 95% and 97%, respectively.

Animal studies conducted in rats showed that more than 25% of the absorbed quercetin is localized in the lung tissue, an added benefit to combat allergy and associated asthma [8]. Addition of quercetin already in the concentration of 100 nM may relax airway rings precontracted with acetylcholine. The cure with quercetin in the concentration of 100 μM avoided a force creation upon exposure to acetylcholine. Moreover, quercetin in the concentration of 50 μM notably augmented isoproterenol-induced relaxations. In addition, dispersion of quercetin, in the concentration of 100 μM, in an *in vivo* model of airways perception notably inhibited methacholine-induced progress in airways resistance [93].

Quercetin inhibits rat tracheal contractility through a presynaptic (involving nitric oxide) and a postsynaptic site of action. The results of Capasso *et al*. [97] study showed that quercetin produced a concentration-dependent inhibition of contractions induced by carbachol. However, quercetin was more active in inhibiting the contractions produced by electrical field stimulation than those induced by carbachol, suggesting a presynaptic site of action (in addition to a postsynaptic effect, as revealed by the inhibitory action of quercetin on carbachol-induced contractions).

Long ago, in the experiment of Johri *et al*. [83], a stabilization effect of quercetin, isolated from onions, on mast cell membrane of rats was shown. In the study of Kimata *et al*. [72], human cultured mast cells were sensitized with IgE, and then treated with flavonoids before challenge with antihuman IgE. Results showed that quercetin inhibited the release of histamine, leukotrienes, prostaglandin D_2_, and granulocyte macrophage-colony stimulating factor from human cultured mast cells in a concentration-dependent manner. Moreover, quercetin inhibited Ca^2+^ influx strongly. The activation of extracellular signal-regulated kinases and c-Jun NH_2_-terminal kinase were clearly suppressed by quercetin. 

Quercetin is effective eosinophilic inflammation suppressor for diseases like allergic rhinitis and asthma. Rogerio *et al.* [91] investigated the anti-inflammatory effect of quercetin and isoquercitrin in a murine model of asthma. In mice feeding with these flavonoids, the amount of white blood cells and eosinophil in the bronchoalveolar lavage fluid, blood and lung parenchyma was detected in lower quantities [91,92].

In another study of Rogerio *et al.* [92], the anti-inflammatory effect of quercetin-loaded microemulsion and quercetin suspension in an experimental model of airways allergic inflammation have been compared. Oral administration of quercetin suspension failed to interfere with leukocyte recruitment, while quercetin-loaded microemulsion inhibited in a dose-dependent way, the eosinophil recruitment to the bronchoalveolar lavage fluid. The microemulsion considerably reduced IL-5 and IL-4 levels too, but has not be capable to interfere with CCL11, IFN-gamma and LTB(4) levels. Oral treatment with microemulsion also decreased the nuclear transcription factor κB activation, and the mucus production in the lung.

Park *et al*. [90] examined whether quercetin could influence eosinophil peroxidase activity. They demonstrated that asthmatic reactions in mice, sensitized by ovalbumin injection, could be notably inhibited by their feeding with quercetin. In another experiment, Sakai-Kashiwabara [100] examined peripheral blood eosinophils and IgE levels after infection by *Mesocestoides corti*. The experiment was designed to examine the influence of quercetin on eosinophil activation induced by SCF stimulation *in vitro*. The addition of quercetin into cell cultures could suppress eosinophil activation induced by SCF stimulation. The minimum concentration of quercetin that caused significant suppression of factor secretion was 5 μM.

Chirumbolo *et al.* [27] studied the anti-allergic effect in the Th1/Th2 immune response. They attempted to determine whether quercetin regulates Th1/Th2 cytokine production, T-bet and GATA binding protein 3 gene expression in ovalbumin-induced asthma model mice. Results of experiment showed that quercetin reduced the increased levels of IL-4, Th2 cytokine production in ovalbumin-sensitized and challenged mice. During the experiment the elevation of interferon-gamma, Th1 cytokine production occurred in quercetin administrated mice. Therefore, quercetin is able to regulate Th1/Th2 balance.

Quercetin is an inhibitor of enzymes responsible for inflammatory reaction, naturally derived PDE4-selective (cyclic nucleotide phosphodiesterases) inhibitor. Chan *et al*. [101] attempted to determine the PDE4(H)/PDE4(L) ratios of different quercetin forms in relation to phosphodiesterase (PDE)-4 activity. The anti-inflammatory effects of PDE4 inhibitors were reported to be associated with inhibition of PDE4 catalytic activity. To reduce inflammation, an inhibition of lipolytic enzyme, human secretory phospholipase A2 group IIA, could be effective as it leads to a decrease in eicosanoids levels. This lipolytic enzyme is therefore of high pharmacological interest in treatment of chronic diseases such as asthma [102]. Good inhibitory activity was shown for quercetin and kaempferol.

Quercetin is structurally related to the anti-allergic drug disodium cromoglycate. Weng *et al*. [82] compared effect of quercetin and cromolyn on cultured human mast cells. Both compounds at the concentration of 100 µM can effectively inhibit secretion of histamine and leukotrienes from primary human cord blood-derived cultured mast cells stimulated by IgE/Anti-IgE. Quercetin is more effective than cromolyn in inhibiting IL-8; reduces IL-6 in a dose-dependent manner; and inhibits cytosolic calcium level increase.

Quercetin is useful in the treatment of immediate (IP), late-phase (LP), and late-late-phase (LLP) asthma responses via inhibition of histamine and protein release, phospholipase A2 activity. It also reduces recruitment of neutrophils and eosinophils into the lung. Jung *et al*. [95] studied this effect for IP and LP on the asthmatic responses in ovalbumin-sensitized conscious guinea pigs. The results of experiment showed that quercetin (7.5 mg/kg) significantly and dose-dependently inhibited immediate and late-phase in asthma responses but less efficiently than dexamethasone and salbutamol. Analogously, Moon *et al*. [96] studied the effects of quercetin inhalation into asthmatic responses by exposure to aerosolized-ovalbumin in conscious sensitized guinea pigs. Results showed that quercetin inhalation decreased IP, LP and LLP in asthma responses, compared with the control. Inhibitory activity of quercetin inhalation on specific airway resistance was similar to effect of its oral administration (10 mg/kg) in asthmatic responses.

### 4.2. Quercetin and Respiratory Allergic Diseases—Epidemiological Evidence

The results of several epidemiological studies suggest that an increase of flavonoid intake is beneficial for asthma [90,103]. Moreover, clinical trials with flavonoids have shown their ameliorative effects on symptoms related to asthma [74]. A protective effect of quercetin consumption on asthma incidence have been demonstrated by epidemiological- and population-based case-control studies in Finland by Knekt *et al*. [104], where higher quercetin intake was associated with lower asthma incidence. In the Netherlands, Willers *et al*. [105] ascertained that consummation of apples during pregnancy may have a protective effect against the development of childhood asthma and allergic diseases. In the Shaheen *et al*. [106] survey of the United Kingdom, about 600 persons with asthma and 900 healthy subjects were investigated due to their diet habits and lifestyle. Total fruit and vegetable intake was only weakly associated with asthma. Apple intake showed a stronger inverse relationship with asthma. This was mostly clear in subjects who consumed at least two apples per week. Onion, tea, and red wine consumption were not related to asthma incidence, suggesting a beneficial effect of apple flavonoids, especially quercetin. 

Tabak *et al*. [107] deduced a positive association between fruit (such as apples) consumption and general pulmonary health. Scientists in their study with over 13,000 individuals proved a positive link of fruit consumption with pulmonary function, probably due to the high quercetin content. However, they also assessed the lack of association between chronic obstructive pulmonary disease (COPD) and flavonol intake. A profitable result of apple consummation on lung function was also demonstrated in another study with about 2500 middle-aged men. More than four apples consumed weekly notably boosted forced expiratory volume compared to subjects who did not eat apples [38,108]. In the study of Woods *et al*. [109] involving 1600 adults in Australia, they discovered that apple and pear intake could be associated with a decreased risk of asthma and a decrease in bronchial hypersensitivity. However, total fruit and vegetable intake is not associated with asthma risk or severity.

### 4.3. Quercetin and Food Allergies

Food allergies are a relevant and common health complaint with an increasing prevalence. Actual allergy therapy is reduced to an avoidance of problematic foods [110]. Studies of this problem are aimed especially at prevention, as the enormous burden is in early childhood [111].

For food allergies, there is a very important practice in an inhibition of dendric cells function. Quercetin together with kaempferol and isoflavones are able to regulate mucosal immunity during hypersensitivity reaction [99].

In the study of Shiseboar *et al*. [98], the effects of quercetin on peanut-induced anaphylactic reactions were investigated in a rat model with rats sensitized by peanut infusion. After daily-intake of quercetin for four weeks, entirely restricted peanut-induced anaphylactic reactions. The comparison with positive control rat group showed that the plasma histamine values in rats with quercetin ingestion were substantially decreased.

Quercetin is therefore a potent suppressor of the on-going immunoglobulin E responses against peanut proteins, and can be introduced as an alternative medicine like a defender against IgE-mediated food allergies.

## 5. Conclusions

Allergic disorders (skin, food and respiratory allergies) have been rapidly increasing worldwide during the last three decades. Therefore, there is a demand for new sources of anti-allergic bioactive compounds. Nowadays, most attention has been focused on flavonoids, especially quercetin. Quercetin displays high antioxidant and anti-inflammatory properties that have been proven by many *in vivo* and *in vitro* studies. Quercetin’s anti-allergic mechanism of action through the inhibition of enzymes and inflammatory mediators has also been extensively studied. It is well known that quercetin is an inhibitor of human mast cell activation through the inhibition of Ca^2+^ influx, histamine, leukotrienes and prostaglandins release. This review also summarizes the role of quercetin in relation to respiratory allergic diseases (*in vitro*, animal and epidemiological studies) and food allergies. The results of the studies prove a unique position of quercetin in the treatment of allergic disorders and the possibility of using phytochemicals such as quercetin for an efficient cure.

## Figures and Tables

**Table 1:** Summarization of quercetin and its anti-allergic effect—*in vitro* studies.

Effect	Studied Models	Mechanism of Action	References
**Inhibition of mast cell activation**	rats	Stabilization of mast cell membrane	Johri *et al.* [83]
		Inhibition of leucotriens release, prostaglandin D2, Ca^2+^ influx	Kimata *et al.* [72]
	Human cultured mast cells	Release of IL-6, IL-8, TNF-2, inhibition of tryptase release, activation of NF-κB	Kempuraj *et al.* [81], Min *et al*. [84]
**Inhibition of histamine release**	*In vitro* malignant cells	Decrease of tryptase, MCP-1, IL-6, histidine decarboxylase (HDC)	Shaik *et al*. [85]
	Antigen sensitized human mast cells	Normal path of Ca^2+^ entry to cells, inhibition of leucotrienes, PGD-2	Fewtrell and Gomperts [86], Kimata *et al*. [72], Weng *et al.* [82]
	Rats mast cells	Release of immunologically induced cells, inhibition of anaphylactic histamine	Haagag *et al*. [87] Pearce *et al*. [88]
	ovalbumin-challenged mice	release from mucosal cells, inhibited eosinophil peroxidase activity and protein content in bronchoalveolar lavage fluid (BALF)	Kaiser *et al*. [89]
**Suppression of eosinophilic inflammation**	OVA-induced asthma model rats	Reduction of eosinophil peroxidase activity, level of IL-4, Th2 cytokine production	Park *et al*. [90] Chirumbolo [27] Jurikova *et al.* [8]
	Murine model of asthma	Decrease in eosinophil counts in bronchoalveolar lavage fluid, inhibition of NF-kappa B	Roger *et al.* [91] Roger *et al.* [92]
**Relaxation of muscles**	Male A/J mice	Isoproterenol induced relaxation	Towsend and Emala [93]
	Smoth muscle murine model of asthma	Reduction of production of inflammatory cytokines	Oliveira *et al*. [94]
	(OVA)-sensitized conscious guinea pigs	Inhibitory activity of quercetin inhalation on sRaw (specific airway resistance)	Jung *et al*. [95] Moon *et al*. [96]
	Isolated tracheal tissue	Concentration-dependent inhibition of contractions induced by both carbachol and electrical field stimulation	Capasso *et al*. [97]
**Suppression of immunoglobulin E against peanuts proteins**	Wister rats	Plasma histamine levels in the quercetin-treated rats were lower significantly, regulate mucosal immunity during hypersensitivity reaction	Shiseboar *et al.* [98] Wei *et al*. [99]
